# Prevalence and characteristics of benign cartilaginous tumours of the knee joint as identified on MRI scans

**DOI:** 10.1186/s40644-023-00572-9

**Published:** 2023-05-25

**Authors:** Johannes Nikolaus Woltsche, Maria Anna Smolle, Dieter Szolar, Marko Bergovec, Andreas Leithner

**Affiliations:** 1 Department of Orthopaedics and Trauma, Medical University of Graz, Graz, Austria; 2 Diagnostikum Graz, Graz, Austria

**Keywords:** Enchondroma, Atypical cartilaginous tumour, Prevalence, Knee joint, MRI

## Abstract

**Background:**

Enchondromas (EC) and atypical cartilaginous tumours (ACT) of the knee joint represent benign/intermediate chondromatous neoplasms of the bone that are most commonly discovered incidentally. Based on small to intermediate-sized cohorts, the prevalence of cartilaginous tumours of the knee as visible in MRI is estimated at 0.2–2.9%. This study aimed at verifying/challenging these numbers via retrospective examination of a larger, uniform patient cohort.

**Methods:**

Between 01.01.2007 and 01.03.2020, 44,762 patients had received an MRI of the knee for any indication at a radiologic centre. Of these, 697 patients presented with MRI reports positive for cartilaginous lesions. In a three-step workflow, 46 patients were excluded by a trained co-author, a radiologist and an orthopaedic oncologist, as wrongly being diagnosed for a cartilage tumour.

**Results:**

Of 44,762 patients, 651 presented with at least one EC/ACT indicating a prevalence of 1.45% for benign/intermediate cartilaginous tumours of the knee joint (EC: 1.4%; ACTs: 0.05%). As 21 patients showed 2 chondromatous lesions, altogether 672 tumours (650 ECs [96.7%] and 22 ACTs [3.3%]) could be analysed in terms of tumour characteristics: With a mean size of 1.6 ± 1.1 cm, most lesions were located in the distal femur (72.9%), in the metaphysis of the respective bone (58.9%) and centrally in the medullary canal (57.4%).

**Conclusions:**

This study revealed an overall prevalence of 1.45% for cartilage lesions around the knee joint. Whilst a constant increase in prevalence was found for ECs over 13.2 years, prevalence remained constant for ACTs.

## Background

Enchondromas (ECs) and atypical cartilaginous tumours (ACTs, formerly known as chondrosarcoma grade 1) are cartilaginous neoplasms located in the medullary canal [1–4]. While ECs represent the most common intramedullary benign cartilaginous lesion [1, 2], ACTs constitute an intermediate neoplasm that is often hard to distinguish from EC [2, 3]. However, distinction between these lesions is of great importance, as ACTs – on the contrary to ECs – require surgical treatment [3, 4]. Certain imaging features allow some assessment of the tumours’ dignity: large lesion size, as well as presence of periosteal reaction, endosteal scalloping or perilesional edema can help differentiate ACT from EC [2, 5]. The latter are most often found in the short bones of the hand (40–65%), whilst long bones (femur, humerus, tibia) are the second most common location for EC, accounting for 25% of cases [6, 7].

ECs typically present clinically silent, with large lesions eventually causing pain or – especially in short bones – pathologic fractures [3, 5]. Therefore, this tumour is most commonly found incidentally during routine clinical imaging (X-ray, CT [computed tomography], MRI [magnetic resonance imaging]). Consequently, the true prevalence of EC remains uncertain [3, 7]. Thus far, five studies have analysed the prevalence of benign cartilaginous lesions around the knee based on MRI, reaching figures between 0.2 and 2.9% [2, 3, 8–10].

As all five studies were based on small to intermediate-sized cohorts, this study aimed at verifying these numbers via retrospective examination of a larger, uniform patient cohort with MRI scans of the knee.

## Methods

### Study design and study population

The current retrospective study was performed with data deriving from a private radiologic centre, performing – apart from numerous other imaging modalities – MRI scans of all body sites. The local medical ethics committee has approved the study (33–630 ex 20/21) that involved patients having received an MRI of the knee for any indication between 01.01.2007 and 01.03.2020. During that time, 44,762 patients had undergone at least one knee MRI scan. Of these, 5182 had undergone MRI scans of both knee joints, resulting in a total of 24,125 and 25,819 MRI scans of the left and right knee, respectively.

All MRI reports of the knee were searched for at least one of the following terms by one of the co-authors (J.W.) who had performed an extensive study of literature and had received intensive training on imaging features of cartilage lesions before data acquisition: “Enchondrom” (enchondroma), “kartilaginäre Läsion” (cartilaginous lesion), “cartilaginäre Läsion” (cartilaginous lesion), “kartilaginärer Tumor” (cartilaginous tumour), “cartilaginärer Tumor” (cartilaginous tumour), “chondrogene Läsion” (chondrogenic lesion), “chondrogener Tumor” (chondrogenic tumour), “Chondrosarkom” (chondrosarcoma), “ACT - atypischer chondromatöser Tumor“ (atypical chondromatous tumour), “atypische chondromatöse Läsion“ (atypical chondromatous lesion).

**Fig. 1:**
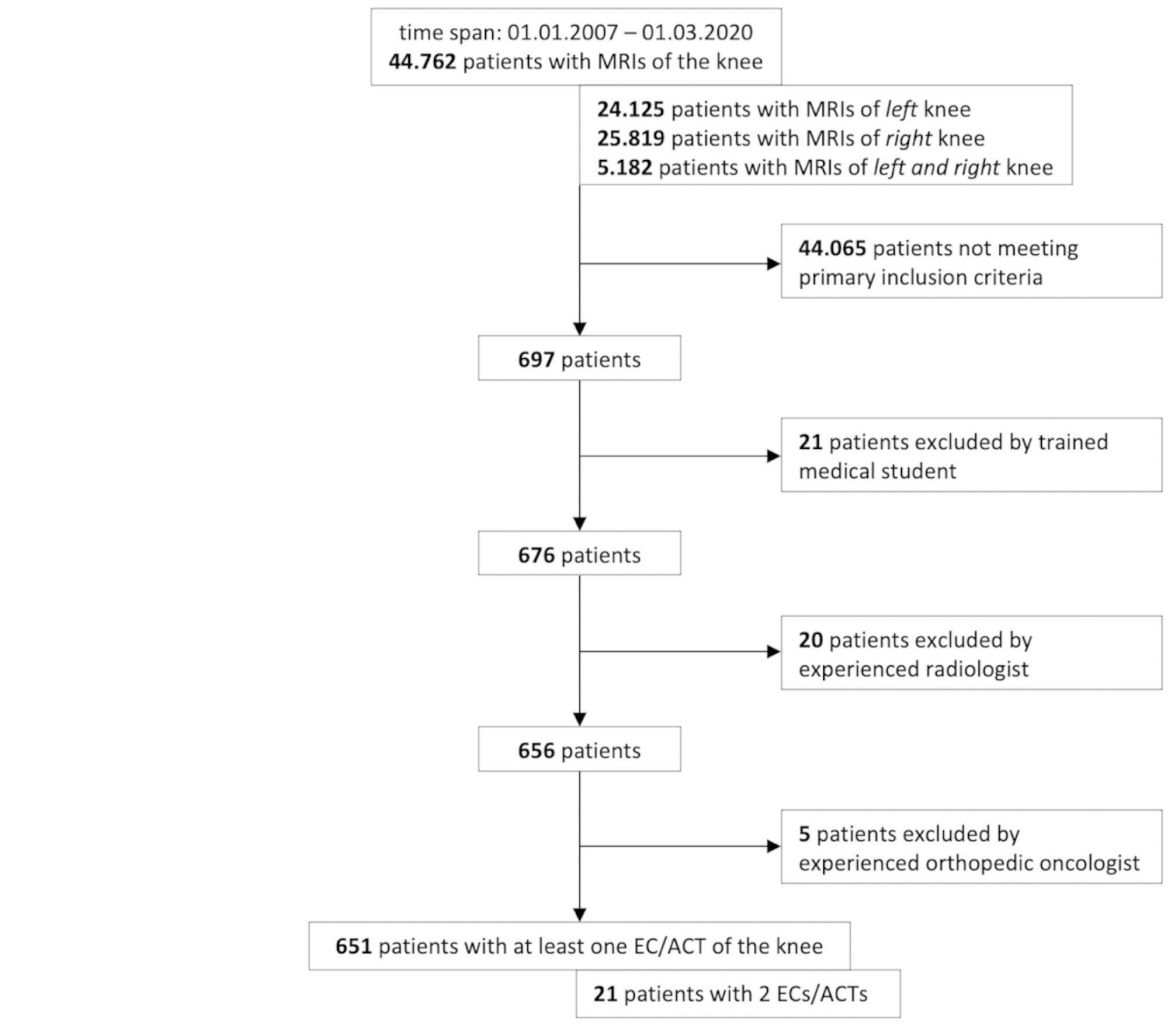
Flow chart representing the filtering of patients with a cartilaginous tumour

Altogether, 697 patients’ MRI reports were positive for at least one search term and were subsequently analysed in further detail (Fig. 1). Of these, 21 patients had to be excluded due to the following reasons: In 11 patients, reason for referral had been a suspected cartilaginous tumour but MRI could not confirm this tentative diagnosis; 3 patients had MRI reports containing not only findings of MRIs of the knee but also of other body regions, in whom they had been diagnosed with a cartilage lesion; 2 patients were initially suspected to have an enchondroma, yet follow-up MRIs led to a change of primary diagnosis; 5 patients had undergone surgical removal of the cartilaginous lesion prior to index imaging.

Reports of the remaining 676 patients were re-examined together with related MRI scans, and a definitive radiologic diagnosis of cartilaginous tumours was confirmed in 635 patients. However, 41 patients had inconclusive reports and images. Therefore, the advice of a senior radiologist was sought for these cases. Thereafter, 20 more patients were excluded, as they did not show typical features of EC/ACT, resulting in 656 patients ultimately eligible.

**Fig. 2:**
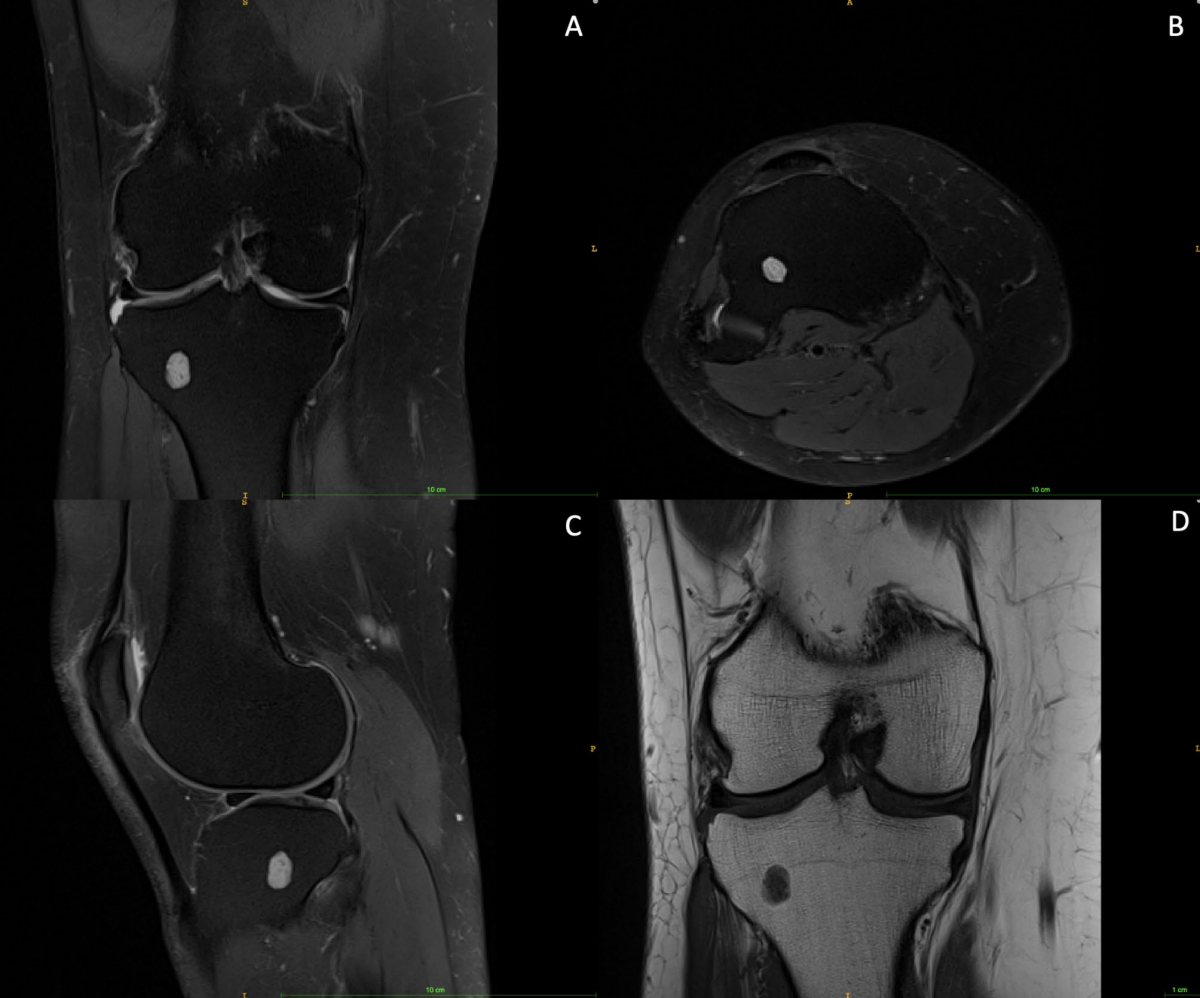
EC of the right tibia: (A) proton density, fat suppression, coronal, turbo spin echo; (B) proton density, fat suppression, transversal, turbo spin echo; (C) proton density, fat suppression, sagittal, turbo spin echo; (D) t1, coronal, turbo spin echo (year of MRI: 2016)

**Fig. 3:**
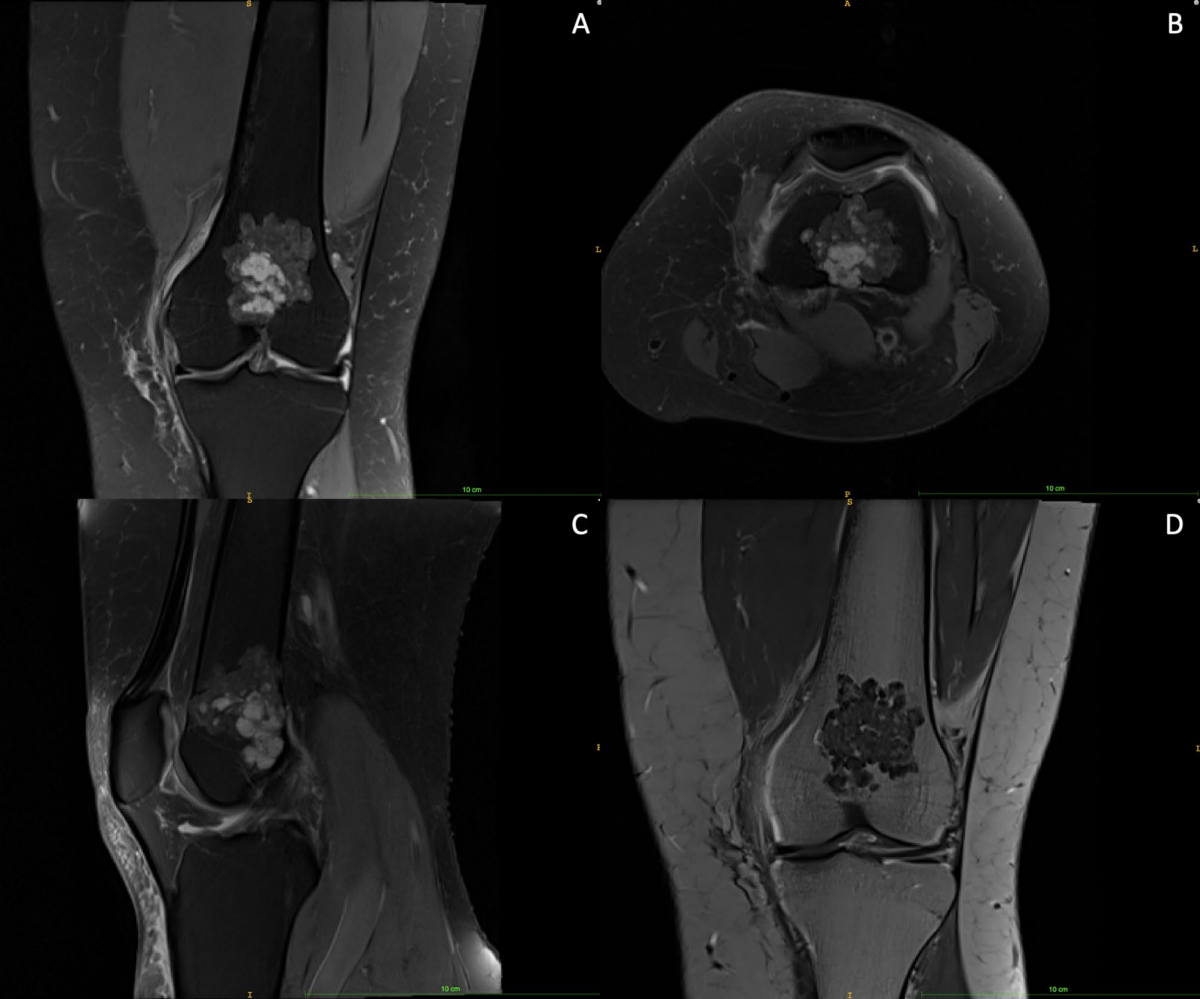
ACT of the left femur: (A) proton density, fat suppression, coronal, turbo spin echo; (B) proton density, fat suppression, transversal, turbo spin echo; (C) proton density, fat suppression, sagittal, turbo spin echo; (D) t1, coronal, turbo spin echo (year of MRI: 2016)

MRI-based differentiation into EC (Fig. 2) and ACT (Fig. 3) was made based on tumour characteristics suspicious of aggressive behavior as proposed by Mulligan et al. [5], Murphey et al. [4], van de Sande et al. [11] and Douis et al. [12], i.e. tumour size > 4.9 cm, periosteal reaction, perilesional edema or deep endosteal scalloping involving ≥ 2/3 of cortical thickness. With either one of these features being positive, chondrogenic lesions were classified as ACT. All tumours exhibiting at least one of these features were thoroughly examined by an experienced orthopedic oncologist, who excluded 5 more patients due to diagnosis of bone infarction (n = 3), postoperative changes (n = 1) and intraosseous ganglion (n = 1).

Overall, 651 patients had a cartilaginous tumour, with 21 patients presenting with 2 cartilaginous lesions at the same time, resulting in 672 cases of EC/ACT.

### Lesion analysis

MRI was considered positive for a cartilaginous tumour on identification of a smooth or lobulated lesion that presented itself as a focal geographic area within the bone marrow, showing low signal intensity on proton-density-weighted and on T1-weighted images and high signal intensity on proton-density fat-suppressed images. Lesions positive for these characteristics but strictly subchondral in location had to be excluded, as they most likely represent different entities such as subchondral cysts, intraosseous ganglia, subchondral edema or contusion.

Apart from patient gender and age, the following tumour-specific features were ascertained: lesion size (maximal tumour diameter in cm), lesion site (femur, tibia, fibula, patella), tumour location (eccentric or central; epiphyseal, epimetaphyseal, metaphyseal, metadiaphyseal, diaphyseal), endosteal scalloping, perilesional edema and periosteal reaction. Furthermore, indication for MRI (tumour associated symptoms or follow-up examinations; no tumour related indication; no documented indication) and whether the patients had undergone dynamic contrast MRI was ascertained.

#### MRI

MRI examinations were performed on two different 3T MRI systems (*Siemens Magnetom Skyra/Siemens Magnetom Vida;* both *Siemens Healthcare Diagnostics GmbH, Austria*) with a 15/18-channel knee coil. Sequences acquired were (1) coronal proton density (PD) with fat suppression (FS) (field of view (VF) 160/140 mm; Matrix (M) 307 × 384/307 × 384; repetition time (TR) 3000/3200 ms; echo time (TE) 34/25 ms; slice thickness (ST) 3/3 mm; interslice gap (IG) 0.6/0.6 mm); (2) transversal PD with FS (VF 160/150 mm; M 307 × 384/307 × 384; TR 5460/4110 ms; TE 37/35 ms; ST 2.5-3/3 mm; IG 0.6/0.6 mm); (3) sagittal PD with FS (VF 160/140 mm; M 307 × 384/307 × 384; TR 2920/2920 ms; TE 34/34 ms; ST 3/3 mm; IG 0.6/0.6 mm); (4) coronal T1 weighted turbo spin echo (TSE) (VF 160/140 mm; M 346 × 384/290 × 484; TR 690/690 ms; TE 11/19 ms; ST 3/3 mm; IG 0.6/0.6 mm; flip angle (FA) 180°/150°).

Gd-DTPA (dose 2 ml/kg body weight) via venous access was administered in 77 of 651 patients (81 of 672 tumours), followed by an MRI examination with the sequence “(4) coronal T1 weighted turbo spin echo” that was subtracted from the native T1 sequence. Additionally, T1 sequences with fat suppression in different dimensions were recorded.

### Statistical analysis

Statistical analyses were carried out with Stata Version 16.1 for Mac (*StataCorp, College Station, Texas, US*). Patient demographics were summarized for based on the total number of patients with cartilaginous lesions. Tumour demographics were summarized from the total number of lesions found in patients. Prevalence was calculated based on the diagnosis of a benign cartilaginous lesion per patient, and not based on the number of cartilage lesions found in total. In line with this, patients without ECs/ACTs undergoing MRI scans of both knee joints during the defined period were counted once only for calculation of prevalence. Normally and non-normally distributed variables were given as means and corresponding standard deviations, as well as medians and corresponding interquartile ranges (IQR), respectively. Fisher’s exact test and t-test were used to assess differences in binary (or ordinary) and continuous variables, respectively, depending on radiological diagnosis. A p-value of < 0.05 was considered statistically significant.

## Results

### Prevalence of benign cartilaginous lesions

**Fig. 4:**
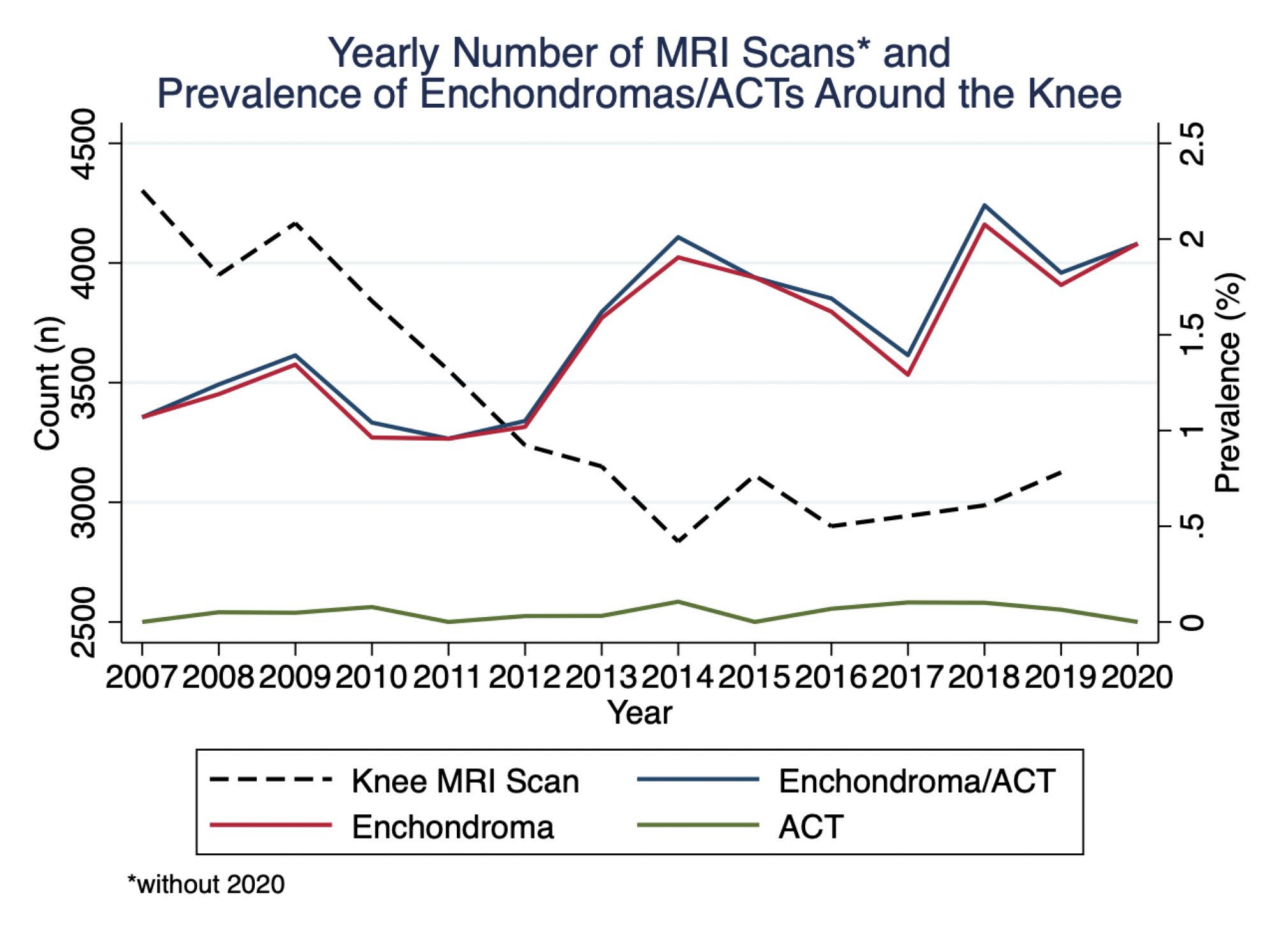
Yearly number of patients with at least one MRI scan (n = 44,762) and estimated prevalence of ECs and ACTs around the knee joint

Between 01.01.07 and 01.03.2020, the prevalence of benign cartilaginous lesions (i.e. ECs and ACTs) amounted to 1.45%. In detail, the separate prevalence for ECs and ACTs was 1.4% and 0.05%, respectively. Over the years, a slight increase in prevalence was observed for ACTs and ECs, as well as ECs only, whereas the prevalence of ACTs did not equally increase (Fig. 4). Notably, the total number of MRI scans performed per year decreased from 2007 to 2014 and remained constant thereafter (Fig. 4).

In 651 (1.45%) out of 44,762 patients with MRI scans of either knee joint taken from 01.01.07 to 01.03.20, a benign cartilaginous lesion was detected incidentally. In 17 of these patients, two lesions in the same knee were found. In additional 4 patients, a cartilaginous lesion was found both in the left and right knee, amounting to 672 tumours in total. Mean age of patients with EC/ACT was 52.1 ± 13.1 years, and 48.4% were males (n = 315). The reason for referral to MRI was suspected EC based on preceding imaging in 79 cases (12.2%), a suspected pathology other than EC in 463 cases (71.1%), and unknown in 109 cases (16.7%).

### Characteristics of cartilaginous lesions

Of the 672 lesions in total, radiological diagnosis was EC in 650 tumours (96.7%), and ACT in 22 lesions (3.3%). Mean tumour size of all cartilaginous lesions was 1.6 ± 1.1 cm. Thirty lesions (4.5%) were sized 4 cm or more, with the largest lesion measuring 9.0 cm (cranio-caudal). The most common location was the distal femur in 490 cases (72.9%), and the majority of lesions was located in the metaphysis (58.9%, n = 396). In relation to the medullary canal, 386 lesions were located centrally (57.4%), and the remaining 286 lesions peripherally (42.6%). Periosteal reaction was present in 2 cases (0.3%), and endosteal scalloping in 15 (2.2%; Table 1).

**Table 1:** Characteristics of benign cartilaginous tumours detected on MRI scan, split by radiological diagnosis of EC vs. ACT (n = 672)

		Total Count (%)	EC (n = 650)	ACT (n = 22)	p-value*
**Tumour size (in cm; mean ± SD)**		1.6 ± 1.1	1.5 ± 0.9	5.1 ± 1.9	< 0.001**
**Side**	Left	312 (46.4)	301 (46.3)	11 (50.0)	0.829
	Right	360 (53.6)	349 (53.7)	11 (50.0)	
**Bone**	Distal femur	490 (72.9)	474 (72.9)	16 (72.7)	**0.011**
	Proximal tibia	137 (20.4)	136 (20.9)	1 (4.6)	
	Proximal fibula	44 (6.6)	39 (6.0)	5 (22.7)	
	Patella	1 (0.1)	1 (0.2)	0 (0.0)	
**Location**	Epiphysis	80 (11.9)	80 (12.3)	0 (0.0)	**< 0.001**
	Epimetaphysis	70 (10.4)	65 (10.0)	5 (22.7)	
	Metaphysis	396 (58.9)	391 (60.2)	5 (22.7)	
	Metadiaphysis	32 (4.8)	27 (4.1)	5 (22.7)	
	Diaphysis	93 (13.9)	86 (13.2)	7 (31.9)	
	Patella	1 (0.1)	1 (0.2)	0 (0.0)	
**Location in Relation to Medullary Canal**	Central	386 (57.4)	383 (58.9)	3 (13.6)	**< 0.001**
	Peripheral	286 (42.6)	267 (41.1)	19 (86.4)	
**Periosteal Reaction**	No	670 (99.7)	650 (100.0)	20 (90.9)	**0.001**
	Yes	2 (0.3)	0 (0.0)	2 (9.1)	
**Medullary Oedema**	No	666 (99.1)	646 (99.4)	20 (90.9)	**0.014**
	Yes	6 (0.9)	4 (0.6)	2 (9.1)	
**Endosteal Scalloping**	No	656 (97.8)	649 (99.9)	8 (36.4)	**< 0.001**
	Yes	15 (2.2)	1*** (0.1)	14 (63.6)	
**Contrast Agent**	No	591 (88.0)	582 (89.5)	9 (40.9)	**< 0.001**
	Yes	81 (12.0)	68 (10.5)	13 (59.1)	

* Fisher’s exact test

** t-test

*** superficial scalloping

All 672 lesions were analysed for radiologic features that allow assessment of tumours dignity [4, 5, 12]. In comparison to ECs, those lesions classified as ACT were significantly larger (p < 0.001), rather located in the proximal fibula (22.7% vs. 6.0%; p = 0.011), and peripherally to the medullary canal (p < 0.001), had been diagnosed via contrast-enhanced MRI (p < 0.001), and presented with periosteal reaction (p = 0.001), medullary oedema (p = 0.014), and endosteal scalloping (p < 0.001; Table 1). Superficial scalloping was present in one EC and 2 ACTs, whereas deep endosteal scalloping was found in 12 ACTs. Notably, endosteal scalloping was present in 11.4% of tumours located in the proximal fibula, compared to 1.5% and 1.6% in the proximal tibia and distal femur, respectively (p = 0.007).

## Discussion

The prevalence of benign cartilage lesions – i.e. enchondromas and ACTs – is difficult to estimate since they seldom cause symptoms and therefore usually present as incidental finding on imaging. In this study, based on 44,762 MRI scans of the knee performed at a single radiology institute within 13.2 years, a prevalence of 1.45% for cartilage lesions around the knee was found.

To the authors knowledge, this is the largest MRI-based study on the prevalence and characteristics of cartilage lesions around the knee. The herein discovered prevalence is lower than the one observed in some previous analyses using MRI scans of the knee. Stomp et al. reported on an estimated population prevalence of 2.8% for cartilage tumours around the knee joint, based on 1,285 MRI scans of the right knee [3]. In the studies by Douis et al. and Walden et al., the prevalence of enchondromas on knee MRIs in children [10] and adults [2], respectively, amounted to both 2.9%. On the other hand, our figure is comparable to the 0.8% (healthy individuals) to 1.5% (knee osteoarthritis patients) reported by Grainger et al. [9]. Yet, with 601 knee MRI scans from healthy individuals and 123 from patients with knee osteoarthritis, the number of scans is nearly 60-times lower than in our cohort.

An even lower prevalence of 0.2% for cartilage lesions around the knee derives from an autopsy study dating back to 1928 [8]. However, this figure has to be interpreted in its historical context, considering that – other than anatomical dissection – MRI scans are highly sensitive in detecting even smallest cartilage lesions, reflected by a mean size of 1.6 cm calculated for all tumours found in the present study.

In our study, only 22 lesions were classified as ACTs amounting to an overall prevalence of 0.05%. This figure is significantly lower than the prevalence of 0.4% reported by Stomp et al. [3]. The discrepancy in ACT prevalence can be explained by the fact that the authors chose a cut-off of 2 cm in lesion’s size to primarily differentiate between enchondromas and ACTs, whilst we used a threshold of 5 cm as proposed by van de Sande et al. [11].

According to van Praag et al., frequency of ACT diagnosis increased between 1989 and 2013, whereas the one of higher grade chondrosarcomas remained constant [13]. Other than in our MRI-based study, the numbers reported by van Praag et al. were based on histopathologically proven ACTs and higher-grade chondrosarcomas [13]. The authors concluded that apart from an ageing population, more frequent imaging and consecutive referral of worrisome cartilage lesions to surgery may have caused the increase in incidence of ACTs [13].

However, in our study, prevalence for ACTs did not increase with time, whilst the one of cartilage lesions in general, and enchondromas in particular, steadily grew from 1.07% to 2007 to 1.8% in 2019 (Fig. 4). Improving image resolution with time and thus increasing sensitivity for even smallest enchondromas can serve as an explanation for this. This hypothesis is also strengthened by the fact that the yearly number of knee MRIs used for final estimation of cartilage lesions’ prevalence even showed a slight decrease with time.

Our findings show that enchondroma represents a frequent and important incidentally diagnosed benign bone tumour. Although prevalence of benign cartilage lesions around the knee joint might have been overestimated by previous studies, this study confirms that enchondroma (1.4%) is an important differential diagnosis of intraosseous lesions around the knee, with a 28 times higher prevalence in comparison to their intermediate counterpart ACT (0.05%).

There are some limitations that should be considered regarding the results of the current study. The herein observed prevalence of ACTs around the knee has to be interpreted with caution, given that differentiation between EC and ACT was MRI based only, and that radiological features indicative of ACT are not utterly defined. Related to this, radiological diagnosis had not been confirmed with histopathology. Given the sampling error associated with differentiation between EC and ACT upon histopathological analysis [11], it is believed that additional histopathology would not have considerably altered results obtained. Another limiting factor is the retrospective design, relying on MRI scans of the knee performed for any reason with no particular focus on detection of cartilaginous lesions. On the other hand, the prevalence of cartilage lesions was estimated based on an abundance of scans with a uniform protocol adopted by the radiology institute where images derived from.

## Conclusions

In summary, the current study revealed an overall prevalence of 1.45% for cartilage lesions around the knee joint. Of all cartilage lesions diagnosed on MRI, 3.3% exhibited features indicative of ACT. Whilst a constant increase in prevalence was found for enchondromas over 13.2 years, prevalence remained constant for ACTs.

## Data Availability

The datasets used and/or analysed during the current study are available from the corresponding author on reasonable request.

## Abbreviations

EC: Enchondroma
ACT: Atypical cartilaginous tumour
PD: Proton density
FS: Fat suppression
VF: Field of view
M: Matrix
TR: Repetition time
TE: Echo time
ST: Slice thickness
IG: Interslice gap
IQR: Interquartile ranges
